# Incidence, susceptibility and outcomes of candidemia in adults living in Calgary, Alberta, Canada (2010–2018)

**DOI:** 10.1186/s12879-023-08050-0

**Published:** 2023-02-20

**Authors:** Samuel Bourassa-Blanchette, Marit M. Biesheuvel, John C. Lam, Alexander Kipp, Deirdre Church, Julie Carson, Bruce Dalton, Michael D. Parkins, Herman W. Barkema, Daniel B. Gregson

**Affiliations:** 1 Department of Medicine Laboratory Medicine, Memorial University, St. John’s, NL Canada; 2 Department of Pathology and Laboratory Medicine, Memorial University, St. John’s, NL Canada; 3 Department of Medicine, Cumming School of Medicine, University of Calgary, Calgary, AB Canada; 4 Department of Pathology and Laboratory Medicine, Division of Microbiology, University of Calgary, Calgary, AB Canada; 5 Faculty of Veterinary Medicine, University of Calgary, Calgary, AB Canada; 6 Division of Infectious Diseases, Department of Medicine, University of California Los Angeles, Los Angeles, CA USA; 7 Alberta Health Services, Pharmacy Services, Calgary, AB Canada; 8 Alberta Health Services, Laboratory Services, #9, 3535 Research Road NW, Calgary, AB T2A 2K8 Canada

**Keywords:** Candida, Incidence, Mortality, Antifungal susceptibility, Fungemia

## Abstract

**Background:**

Candidemia is increasing in frequency and is associated with high mortality. We sought to determine the burden of illness, the population it affects and its resistance profile in our region.

**Methods:**

The Calgary Zone (CZ) provides all care for residents of Calgary and surrounding communities (~ 1.69 million) via five tertiary hospitals each served by a common single laboratory for acute care microbiology. All adult patients in the CZ with at least one *Candida* spp.-positive blood culture between January 1, 2010, and December 31, 2018, were identified using microbiological data from Calgary Lab Services, the laboratory that processes > 95% of all blood culture samples in the CZ, were reviewed for the study.

**Results:**

The overall annual incidence of candidemia among individuals living in the CZ was 3.8 per 100,000 persons (Median age 61 years (IQR 48–72) and 221/455 (47.4%) were female). *C. albicans* was the most common species (50.6%), followed by *C. glabrata,* (24.0%). No other species accounted for more than 7% of cases. Overall mortality at 30, 90, and 365 days was 32.2, 40.1, and 48.1% respectively. Mortality rate did not differ by *Candida* species. Of individuals who developed candidemia, more than 50% died within the next year. No new resistance pattern has emerged in the most common *Candida* species in Calgary, Alberta.

**Conclusions:**

In Calgary, Alberta, the incidence of candidemia has not increased in the last decade. *C. albicans* was the most common species and it remains susceptible to fluconazole.

## Background

Candidemia is a common cause of hospital-acquired bloodstream infection, with ~ 22,660 cases of candidemia annually in the United States and an estimated overall annual incidence of 7.0 cases per 100,000 persons [1–3]. Increased medical devices, immunosuppression and broad-spectrum antimicrobials have increased the incidence [4]. Mortality can exceed 40%, with marginal improvement over the last decade despite novel antifungals [5–8]. Hospitalizations complicated by invasive candidiasis cost > $1 billion USD/year in the USA [9].

A shift from *Candida albicans* to *Candida* non-*albicans* spp. fungemia has increased infections resistant to first-line azoles with global emergence of *Candida auris* causing concerns regarding antifungal resistance and increased mortality [10–12]. The Infectious Diseases Society of America (IDSA) published its inaugural recommendations for treating candidiasis in 2000, followed by three updates, primarily to describe newer and less toxic systemic antifungals (i.e., echinocandins).

Current objectives were to characterize: (1) the burden of candidemia in Calgary; (2) those that develop candidemia; and (3) Candida antimicrobial resistance (AMR) from 2010 to 2018.

## Methods

### Study setting and data sources

The Calgary Zone (CZ) provides comprehensive medical and surgical care to 1.69 million individuals in Calgary and surrounding communities, in Alberta, Canada, except for acute heart, liver and lung surgical transplants. We report a 9-year population-based study of candidemia epidemiology. The CZ includes four medical centres: Foothills Medical Centre, Peter Lougheed Centre, Rockyview General Hospital, and South Health Campus. Patient characteristics and medical information were retrieved (Sunrise Clinical Manager, Discharge Abstract Database, National Ambulatory Care Reporting System, Provincial Registry and Pharmaceutical Information Network). All patients were assigned a unique code to ensure anonymity, data were stored on a secure University of Calgary (UofC) with double password protection and accessed only by study investigators. The Conjoint Health Research Ethics Board approved the study with a waiver for informed consent (REB18-1659).

### Patients

All adults (age ≥ 18) in the CZ with at least one *Candida* spp.-positive blood culture from January 1, 2010, to December 31, 2018, were included, based on microbiological data from Calgary Lab Services, the laboratory that processes > 95% of all blood culture samples. The index blood culture and onset of candidemia was defined as the first positive blood culture. Data were merged on unique ID and candidemia characteristics (source of infection, duration, complications, treatments, etc.). Multiple episodes with the same *Candida* species were considered separate events if there was at least 30 days between occurrences and blood culture cleared between episodes. Duration of candidemia was defined as the interval from the last positive blood culture to the first negative culture. Clearance was documented when there was a negative blood culture for *Candida* not followed by further blood cultures containing it. Chart reviews ensured the first registered candidemia episode from January 2010 to March 2010 was unrelated to a previous event. Admission data and risk factors for candidemia were recorded. Electronic Medical Records (EMR) were reviewed for each patient by infectious disease clinicians (SBB, JCL and DBG). Community-onset candidemia which includes healthcare-associated and community-acquired candidemia was defined if the organism was isolated within 48 h after initial hospitalization and not attributable to prior hospitalization. Recurrence of infection was established based on review of available information from the EMR.

Detailed chart reviews were conducted, capturing patient demographics and comorbidities, disposition of hospitalization, biochemical and microbiology data at incident blood culture draw, investigations, metastatic phenomenon, antimicrobial and immunosuppressive usage, and clinical outcomes. For patients transferred to the CZ, postal code was used to verify CZ residence. The CZ includes four hospitals with one site acting as the centre for all transplantation within the zone consisting of hematological and renal transplantation only. Each of the hospitals is equipped with an intensive care unit.

### Laboratory analyses

Blood cultures were incubated for 5 d in BacTAlert™ FA and FN blood culture bottles (BioMerieux, Montreal, Canada) [13]. From 2010 to 2014, on-site identification used germ tube testing, morphology on Cornmeal Blue Agar and Vitek 2 YST ID card (BioMérieux, Montreal, QC, Canada) or API-20C AUX System (BioMérieux, Montreal, QC, Canada) [14, 15]. Thereafter, identification was primarily matrix-assisted laser desorption/ionization Vitek-MS™ (BioMérieux, Montreal, QC, Canada), plus phenotypic identification and Vitek 2 YST ID cards when MALDI-TOF did not provide identification. Sensititre YeastOne Y-05 colorimetric broth microdilution panels (Trek Diagnostic Systems Inc, Cleveland, OH, USA) were used for susceptibility testing, following package insert and Clinical Laboratory Standard Institute (CLSI) M60 protocols with supplementation from Pfaller et al. if the *Candida* species lacked CLSI breakpoint or epidemiological cut-off value (ECV) [16–18]. For epidemiological cut-offs, *Candida* isolates were labelled as wild type (WT), or non-wild type (NWT) based on CLSI definition [17].

### Statistical analyses

Regional dashboard data from Alberta.ca were used to determine the CZ population to calculate annual incidence rates [19]. Differences in the incidence of the six most common *Candida* species were tested using a Poisson regression model, with number of candidemia patients as the outcome variable and year as a continuous explanatory variable and annual total population size added as exposure.

Differences between hospital- and community-onset candidemia regarding occurrence of co-morbidities were tested using equality of proportions. A multilevel logistic regression model was used, with isolates classified susceptible or resistant. Hospital site was a fixed effect to correct for patients within a hospital who are potentially more alike in terms of antimicrobial susceptibility than patients between hospitals.

Normally distributed variables were reported as means with standard deviations (SD) whereas non-normally distributed data were reported as medians with inter-quartile ranges (IQR). All data manipulations and analyses used STATA (Stata Corp, 2015, Stata: release 16). Statistical significance was P ≤ 0.05.

## Results

### Incidence

**Fig. 1:**
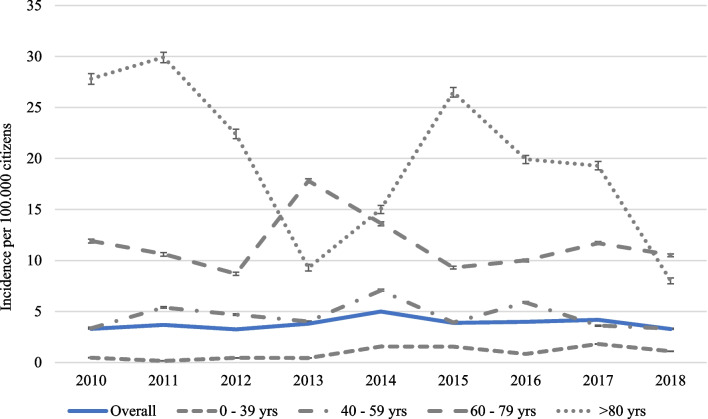
Overall annual incidence of candidemia per 100,000 citizens among individuals in the Calgary Health Region, and the annual incidence per 100,000 citizens among individuals 0–39 years, 40–59 years, 60–79 years and > 80 years between 2010 and 2018

Overall annual incidence of candidemia among patients living in the CZ was 3.8 per 100,000 population, ranging from 3.2 per 100,000 in 2012 to 5.0 per 100,000 in 2014 (P < 0.001; Fig. 1). Incidence changed throughout the study but did not differ between start and end (*P* = 0.54). The annual incidence among patients between 0 and 39 years was generally low, ranging between 0.2 and 1.8 per 100,000, and increased during the study period (*P* < 0.01) (Fig. 1). The annual incidence among patients between 40 and 59 years was steadily low and ranged between 3.3 and 7.1 per 100,000. The annual incidence among patients between 60 and 79 years and > 80 years fluctuated more and was higher, ranging between 8.7 and 17.8 per 100,000 and 8.0–29.9 per 100,000, with no significant changes over the study period.

### Patient demographics

In total, 455 individuals with 466 episodes of candidemia were analysed. Patients with multiple episodes had events separated by 692 days (IQR 403–852). Median age was 61 years (IQR 48–72), ranging from 19 to 100 years, and 47.4% of the patients were female (Table 1). Median hospital stay was 28 d (IQR: 14–55 d), ranging from 0 to 1470 d, with 53.7% getting ICU support. In total, 16.7% of patients were transferred to CZ from another hospital; based on postal code, 44 non-Calgary residents were excluded from incidence calculations. Most patients were discharged from the hospital (54.9%), 42.1% died before discharge and some left the hospital against medical advice (2.6%) or were transferred to another hospital (0.2%). Most (n = 366, 78.5%) patients were treated with antimicrobials 30 d before candidemia, approximately half (48.5%) had cardiovascular disease as a comorbidity and approximately one-quarter had diabetes, chronic liver disease, pulmonary disease, or cancer (Table 1).

**Table 1:** Characteristics of individuals with candidemia in the Calgary zone from 2010 to 2018 (n = 455)

Characteristics	No. (%)
Mean age (SD)	59 (17^1^)
Female	221 (47.4)
Comorbidities	
Diabetes	112 (24.0)
Cardiovascular disease	226 (48.5)
Ischemic heart disease	82 (17.6)
Congestive heart failure	68 (14.6)
Arrythmia	110 (23.6)
Congenital (incl. BAV)	2 (0.4)
Intracardiac device	5 (1.1)
Peripheral vascular disease	24 (5.2)
Cerebrovascular disease	32 (6.9)
Preceding bacterial infective endocarditis	19 (4.1)
Chronic liver disease	91 (19.5)
Cirrhosis fibrosis	28 (6.0)
Hepatitis C	50 (10.7)
Hepatitis B	2 (0.4)
Alcohol liver disease	24 (5.2)
Autoimmune hepatitis	8 (1.7)
Liver transplant	2 (0.4)
Pancreatitis	19 (4.1)
Chronic kidney disease	76 (16.3)
Haemodialysis	22 (4.7)
Peritoneal dialysis	8 (1.7)
Pulmonary disease	97 (20.8)
Bronchitis (incl. COPD)	59 (12.7)
Asthma	24 (5.2)
Obstructive sleep apnoea	7 (1.5)
Other factors	
Cancer in last year	108 (23.2)
Solid organ cancer	68 (14.6)
Blood cancer	33 (7.1)
Solid organ transplantation	17 (3.7)
Individuals receiving parenteral nutrition (TPN)	76 (16.3)
Patient received ≥ 10 mg corticosteroids per day^2^ for last 30 d	125 (26.8)

^1^Standard deviation

^2^Equivalent dose of ≥ 10 mg prednisone

### Candidemia

Approximately 23.4% of *Candida* bloodstream infections were community acquired, with the remainder hospital acquired. Overall, patient demographics were similar between groups, although patients with hospital-acquired candidemia were more likely to have cardiovascular disease (51.3 vs. 39.5%; P = 0.03), specifically congestive heart failure (17.7 vs. 4.6%; P < 0.001) and arrhythmia (27.5 vs. 11.0%; P < 0.01), but less likely to have peripheral vascular disease (3.6 vs. 10.0%; P = 0.01). In addition, hospital-onset candidemia more often had cancer in the last year (25.8 vs. 14.7%; P = 0.02), were receiving parenteral nutrition (20.7 vs. 1.8%; P < 0.001) or received ≥ 10 mg prednisone equivalent of corticosteroids daily for the last 30 days (30.3 vs. 15.6%; P < 0.01)[20]. Asthma was more apparent in community-onset candidemia infections (9.2 vs. 3.9%; P = 0.03) (Table 1).

**Fig. 2:**
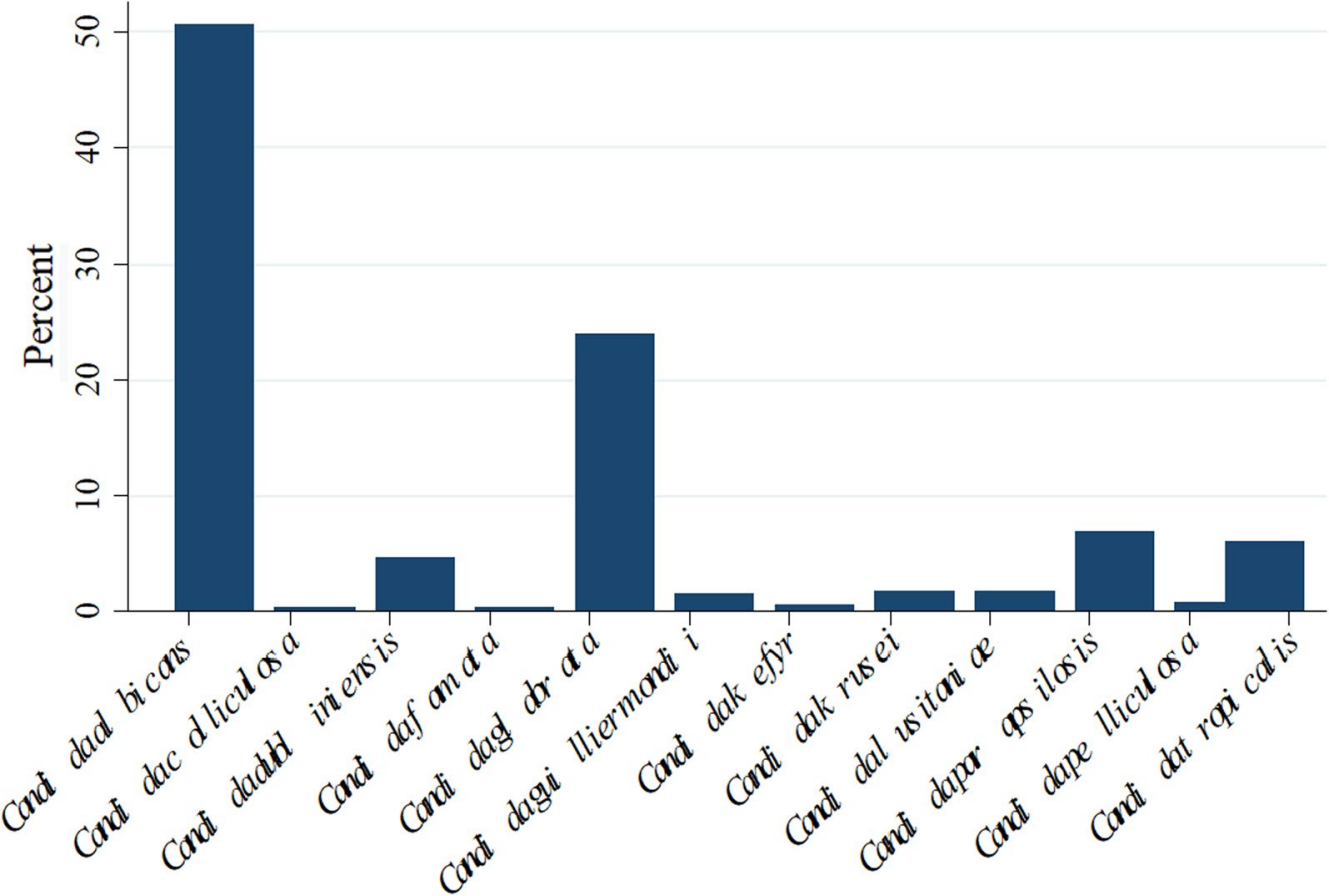
Distribution of species causing candidemia between 2010 and 2018

**Fig. 3:**
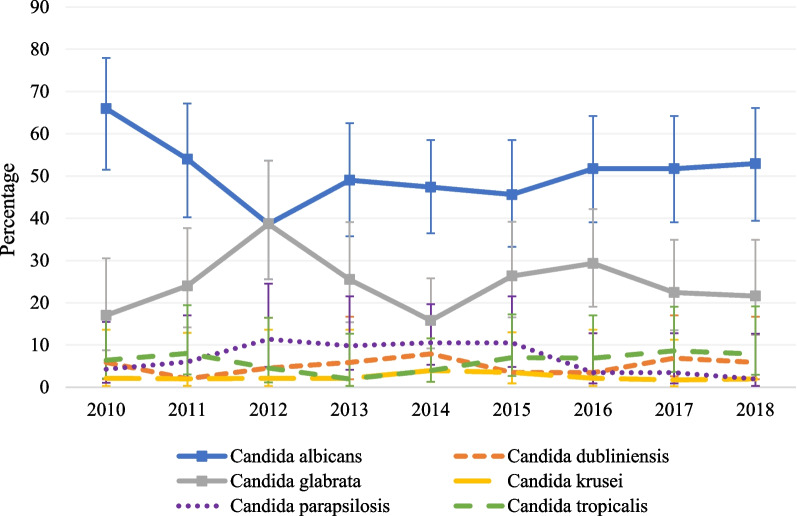
Relative proportion with 95% confidence intervals of *Candida* species causing *Candida* bloodstream infection each year, over time between 2010 and 2018

The most common isolated species were *C. albicans* (50.6%) and *C. glabrata* (24.0%) (Fig. 2). An infection with a second and different species of *Candida* isolated > 2 d after the first positive culture occurred in 5.8% of the cases. In 32% of these cases, patients got an infection with *C. albicans* and *C. glabrata*. No other trends were observed. The relative proportion of the six most common *Candida* species (*C. albicans*, *C. glabrata*, *C. parapsilosis*, *C. dubliniensis*, *C. krusei* and *C. tropicalis*) did not change over time, apart from a transient decrease in *C. albicans* between 2010 and 2012 (*P* = 0.02) and a significant increase in *C. glabrata* infections (*P* = 0.03). (Fig. 3).

### Mortality

Forty-four (9.4%) of patients with candidemia died within 24 h after the index blood culture and were excluded from analyses. For the remaining 422 patients, all-cause mortality rate at 30 d, 90 d and 1 y post candidemia was 32.2% (95% CI: 27.7–36.7%), 40.1% (95% CI: 35.4–44.8%) and 48.1% (95% CI: 43.3–52.9%), respectively. The all-cause mortality rate at 30 d, 90 d and 1 y post candidemia was generally higher in the patient group with hospital acquired candidemia infections than the community-onset group (35.7 vs. 21.0%, *P* < 0.01; 42.6 vs. 32.0%, *P* = 0.06, and 51.9 vs. 36.0%, *P* < 0.01)*.* When stratifying mortality by the three most common *Candida* species*,* the all-cause mortality rate at 30 d, 90 d and 1 year post candidemia did not differ from the overall rate. For *C. albicans* (n = 218 patients), mortality at 30 d, 90 d and 1 year were 32.1% (95% CI: 25.9–38.3%), 38.5% (95% CI: 32.0–45.0%) and 45.4% (95% CI: 38.8–52.0%), respectively. For *C. glabrata* (n = 106 patients) mortality at 30 d, 90 d and 1 year were 34.0% (95% CI: 25.0–43.0%), 43.4% (95% CI: 34.0–52.8%) and 55.7% (95% CI: 46.2–65.2%). Finally, for *C. parapsilosis*, mortality at 30 d, 90 d and 1 year were 26.5% (95% CI: 11.7–41.3%), 38.2% (95% CI: 21.9–54.5%) and 44.1% (95% CI: 27.4–60.8%).

**Fig. 4:**
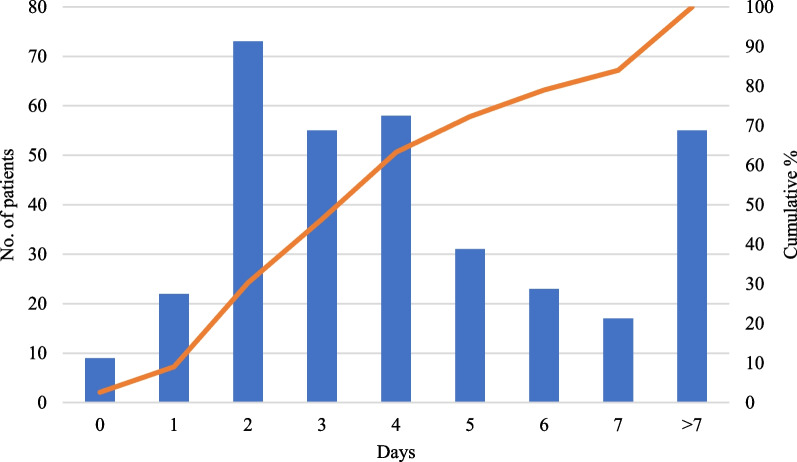
Length in days between first positive and first negative culture for 343 patients with documented clearance of *Candida* spp. from blood

Documented blood culture clearance of *Candida* spp*.* from blood occurred in 343 patients, with a median duration of candidemia of 4 d (IQR 2–6 d). Furthermore, 84% of patients cleared their Candidemia in < 7 days (Fig. 4).

Average interval from hospital admission to candidemia diagnosis was 16 d (95% CI: 13–20 d) with a median of 8 d. Readmission within 1 y occurred in 102 patients (21.9%), but this was due to candidemia in only 5 patients (4.9%).

Nine (2.0%) patients had two or more distinct episodes of candidemia during the study period but were classified as unrelated. Most were admitted twice, and 2 were re-admitted 3 times. For these 9 patients, median interval from discharge to readmission was 599 d (IQR 403–852 d), ranging from 48 to 2,026 d.

### Antifungal susceptibilities of *Candida* species causing candidemia

Susceptibility testing was performed for all isolates of *C. albicans* (n = 242), *C. glabrata* (n = 117), *C. parapsilosis* (n = 33) and *C. tropicalis* (n = 30). Resistance to common antifungals ranged from 0 to 2.5%, and percentage of non-wild types from 0.0 to 9.4% for *C. albicans* (Table 2)*.* These percentages were slightly higher for *C. glabrata* (1.0–9.4% and 0.0–7.8%) (*P* < 0.01; *P* = 0.63; Table 3) and higher for *C. tropicalis* isolates ((0.0–31.0% and 0.0–52.9%) (*P* < 0.001; *P* < 0.001; Table 4)). No resistance was observed for *C. parapsilosis* (Table 5). No difference in percentages of susceptible dose dependent (SDD and resistant isolates was observed among the 4 hospitals, except C. *glabrata* isolates resistant for fluconazole decreased from 5.1 (95% CI: 1.9–10.8%) to 1.2% (95 CI: 0.0–46.9%) after removing one site from the equation (this site had much higher local resistance than the other three sites).

**Table 2:** Antifungal susceptibilities of 242 *Candida albicans* isolates

Methodology	Antifungal	No.	MIC^50^	MIC^90^	Susceptible	Resistant	
			(µg/mL)	(µg/mL)	(%)	(%)	95% CI
CLSI	Caspofungin	241	0.06	0.12	99.6	0.4	0.0–2.3
Breakpoints	Micafungin	190	0.008	0.016	100	0	0.0–1.9
	Voriconazole	241	0.008	0.015	97.9	2.1	0.7–4.8
	Fluconazole	242	0.5	1	97.5	2.5	0.9–5.3

		No.	MIC^50^	MIC^90^	WT^1^	NWT^2^	95% CI
			(µg/mL)	(µg/mL)	(%)	(%)	
CLSI	Amphotericin B	242	0.5	0.5	100	0	0.0–1.5
ECV^3^	Posaconazole	125	0.03	0.06	90.5	9.5	5.1–16.2

^1^Wild type

^2^Non-wild type

^3^Epidemiological cut-off value

**Table 3:** Antifungal susceptibilities of 117 *Candida glabrata* isolates

Methodology	Antifungal	No.	MIC^50^	MIC^90^	Susceptible	Resistant	
			(µg/mL)	(µg/mL)	(%)	(%)	95% CI
CLSI	Caspofungin	117	0.06	0.12	90.6	9.4	4.8–16.2
Breakpoints	Micafungin	102	0.016	0.016	99.0	1.0	0.0–5.3
	Fluconazole	117	4	32	94.9	5.1	1.9–10.8

		No.	MIC^50^	MIC^90^	WT^1^	NWT^2^	95% CI
			(µg/mL)	(µg/mL)	(%)	(%)	
CLSI	Amphotericin B	117	0.5	0.5	100.0	0.0	0.0–3.1
ECV^3^	Itraconazole	60	0.25	0.5	98.3	1.7	0.0–8.9
	Posaconazole	63	0.25	1	93.7	6.3	1.8–15.5
	Voriconazole	116	0.06	0.25	92.2	7.8	3.6–14.2

^1^Wild type

^2^Non-wild type

^3^Epidemiological cut-off value

**Table 4:** Antifungal susceptibilities of 30 *Candida tropicalis* isolates

Methodology	Antifungal	No.	MIC^50^	MIC^90^	Susceptible	Resistant	95% CI
			(µg/mL)	(µg/mL)	(%)	(%)	
CLSI	Caspofungin	30	0.12	0.25	90.0	10.0	2.1–26.5
Breakpoints	Micafungin	26	0.03	0.06	100.0	0.0	0.0–13.2
	Fluconazole	30	2	> 256	69.0	31.0	14.7–49.4
	Voriconazole	30	0.12	16	70.4	29.6	12.3–45.9

		No.	MIC^50^	MIC^90^	WT^1^	NWT^2^	95% CI
			(µg/mL)	(µg/mL)	(%)	(%)	
CLSI	Amphotericin B	30	0.5	1	100.0	0.0	0.0–11.6
ECV^3^	Itraconazole	18	0.25	256	77.8	22.2	6.4–47.6
	Posaconazole	17	0.25	16	47.1	52.9	27.8–77.0

^1^Wild type

^2^Non-wild type

^3^Epidemiological cut-off value

**Table 5:** Antifungal susceptibilities of 33 *Candida parapsilosis* isolates

Methodology	Antifungal	No.	MIC^50^	MIC^90^	Susceptible	Resistant	
			(µg/mL)	(µg/mL)	(%)	(%)	95% CI
CLSI	Caspofungin	33	0.5	0.5	100.0	0.0	0.0–10.6
Breakpoints	Micafungin	29	1	2	100.0	0.0	0.0–11.9
	Fluconazole	33	0.5	1	100.0	0.0	0.0–10.6
	Voriconazole	33	0.008	0.015	100.0	0.0	0.0–10.6

		No.	MIC^50^	MIC^90^	WT^1^	NWT^2^	95% CI
			(µg/mL)	(µg/mL)	(%)	(%)	
CLSI	Amphotericin B	33	0.5	0.5	100.0	0.0	0.0–10.6
ECV^3^	Posaconazole	15	0.03	0.06	100.0	0.0	0.0–21.8

^1^Wild type

^2^Non-wild type

^3^Epidemiological cut-off value

## Discussion

Candidemia remains a significant cause of morbidity and mortality [21]; the incidence in the CZ did not change from 2010 to 2018, but it was higher than in 1999 to 2004. [4] In this study, there was a significant difference between mortality in community acquired candidemia and hospital acquired candidemia. These groups of patients differed with patients suffering from hospital acquired candidemia more likely having cancer, receiving parenteral nutrition or having been exposed to steroids. These markers of frailty may explain in part while this group’s mortality was higher. Parenteral nutrition and malignancy were found to be risk factors for candidemia from *C. tropicalis* in the past [21]. The overall incidence of *Candida* infections continues to be lower than in other parts of the world [22–24]. *Candida albicans* remains the most common species causing candidemia followed by *C. glabrata*.[4] However, the proportion of individuals with *C. glabrata* and its resistance to fluconazole remain relatively low [3, 25]. Furthermore, Alberta has one of the youngest populations in Canada, whereas areas with a higher median age have higher rates of Candidemia [26]. In our study, less recurrence of candidemia occurred compared to a recently published population-based US contemporary cohort [26].

Regarding the discrepancy between echinocandin resistance when comparing micafungin and caspofungin susceptibility profiles for *C. glabrata* and *C. tropicalis*, our laboratory stopped reporting caspofungin at the beginning of the last decade due to unreliability in intermediate or resistant results [18]. Thereafter, micafungin susceptibility was used as a surrogate [27]. The *C. tropicalis* isolates in our study had over 20% resistance to fluconazole. In our study, fluconazole resistance in *C. tropicalis* was higher than expected. This is consistent with the findings of a Spanish study [28]. In this cohort of patients with *C. tropicalis* candidemia, 23% of isolates were fluconazole resistance. Their finding and ours suggests possible horizontal transmission within a high-risk population. Resistance to azoles in other settings for this *Candida* species has been reported to be associated with over expression of ERG11 [29]. Other studies identified solid organ transplantation as a main risk factor for increased candidemia [30]. That acute solid organ transplant surgery and immediate post-operative recovery do not occur in the CZ may artificially lower the incidence. The transient change in proportion of *C. albicans* was attributed to noise.

Isolates of *C. albicans*, *C. parapsilosis* and *C. glabrata* within the CZ continue to be susceptible to fluconazole, although *C. tropicalis* which was not reliably susceptible to azoles. Preserved susceptibility of *C. parapsilosis* and *C. glabrata* to fluconazole is not reported in other regions, including the United States [31].

Unlike other studies, we did not exclude patients with more than 1 episode when those episodes were separate (demonstrated blood clearance with at least 30 d between episodes) [32]. We felt that the expertise of the infectious disease specialist would allow for proper determination if repeat episodes of candidemia occurred in the same patient were associated with a single episode or separate events. Regardless, the impact was minimal, as only 7 patients had > 1 candidemia episode.

Several limitations are acknowledged. No acute transplant surgical care within the CZ may underestimate the population incidence of candidemia. Incomplete data within the EMR did not allow for calculating a Charlson comorbidity index to adjust for the lack of acute transplant surgical care within the CZ.

The length of candidemia could not always be determined as follow up blood cultures to document clearance were not always collected in relation to their bloodstream infection event. Furthermore, some first negative blood cultures were collected later than recommended by clinical practice guidelines, potentially increasing the duration of the candidemia without altering severity of the infection [33]. However, in 87% of patients the interval from their last positive to their first negative culture was < 3 d, so the effect was likely small.

In summary, in the largest Canadian study on candidemia, incidence was higher than previously measured, but did not increase through the duration of the study. Short- and long-term mortality from candidemia continue to be high. In our study population, resistance to antifungal for *C. albicans* has not emerged.

## Data Availability

The anonymized data are stored securely on the University of Calgary cloud platform It can be made available through reasonable request to corresponding author.

## References

[1] Zaoutis TE, Argon J, Chu J, Berlin JA, Walsh TJ, Feudtner C (2005). The epidemiology and attributable outcomes of candidemia in adults and children hospitalized in the United States: a propensity analysis. Clin Infect Dis.

[2] Pfaller MA, Diekema DJ, Jones RN, Sader HS, Fluit AC, Hollis RJ (2001). International surveillance of bloodstream infections due to Candida species: frequency of occurrence and in vitro susceptibilities to fluconazole, ravuconazole, and voriconazole of isolates collected from 1997 through 1999 in the SENTRY Antimicrobial Surv. J Clin Microbiol.

[3] Tsay SV, Mu Y, Williams S, Epson E, Nadle J, Bamberg WM, et al. Burden of Candidemia in the United States, 2017. Clin Infect Dis. 2020;e449–e453. 10.1093/cid/ciaa193 32107534

[4] Laupland KB, Gregson DB, Church DL, Ross T, Elsayed S (2005). Invasive Candida species infections: a 5 year population-based assessment. J Antimicrob Chemother.

[5] Corzo-Leon DE, Alvarado-Matute T, Colombo AL, Cornejo-Juarez P, Cortes J, Echevarria JI (2014). Surveillance of Candida spp bloodstream infections: epidemiological trends and risk factors of death in two Mexican tertiary care hospitals. PLoS ONE.

[6] Al-Rawahi GN, Roscoe DL (2013). Ten-year review of candidemia in a Canadian tertiary care centre: predominance of non-albicans Candida species. Can J Infect Dis Med Microbiol.

[7] Raja NS (2021). Epidemiology, risk factors, treatment and outcome of Candida bloodstream infections because of *Candida albicans* and Candida non-albicans in two district general hospitals in the United Kingdom. Int J Clin Pract.

[8] MacPhail GLP, Taylor GD, Buchanan-Chell M, Ross C, Wilson S, Kureishi A (2002). Epidemiology, treatment and outcome of candidemia: a five-year review at three Canadian hospitals. Mycoses.

[9] Benedict K, Jackson BR, Chiller T, Beer KD (2019). Estimation of direct healthcare costs of fungal diseases in the United States. Clin Infect Dis.

[10] Spivak ES, Hanson KE. Candida auris: an emerging fungal pathogen. J Clin Microbiol. 2018;56. 10.1128/JCM.01588-17 PMC5786713 29167291

[11] Kim EJ, Lee E, Kwak YG, Yoo HM, Choi JY, Kim SR (2020). Trends in the epidemiology of candidemia in intensive care units from 2006 to 2017: results from the Korean National Healthcare-Associated Infections Surveillance System. Front Med (Lausanne).

[12] Horn DL, Neofytos D, Anaissie EJ, Fishman JA, Steinbach WJ, Olyaei AJ (2009). Epidemiology and outcomes of Candidemia in 2019 patients: data from the Prospective Antifungal Therapy Alliance Registry. Clin Infect Dis.

[13] Ericson EL, Klingspor L, Ullberg M, Özenci V (2012). Clinical comparison of the Bactec Mycosis IC/F, BacT/Alert FA, and BacT/Alert FN blood culture vials for the detection of candidemia. Diagn Microbiol Infect Dis.

[14] Garcia LS (2010). Clinical microbiology procedures handbook.

[15] Leber AL (2016). Clinical microbiology procedures handbook.

[16] Pfaller MA, Diekema DJ (2012). Progress in antifungal susceptibility testing of *Candida* spp. by use of Clinical and Laboratory Standards Institute broth microdilution methods, 2010 to 2012. J Clin Microbiol.

[17] CLSI. Epidemiological cutoff values for antifungal susceptibility testing, 3rd edn. Clinical and Laboratory Standards Institute. 2020.

[18] CLSI. Performance standards for antifungal susceptibility testing of yeasts, 2nd edn. Clinical and Laboratory Standards Institute. 2020.

[19] Calgary—Population. 2022. https://regionaldashboard.alberta.ca/region/calgary/population/#/?from=2001&to=2019. Accessed 28 Aug 2022.

[20] McCarty TP, White CM, Pappas PG (2021). Candidemia and invasive Candidiasis. Infect Dis Clin N Am.

[21] Muñoz P, Giannella M, Fanciulli C, Guinea J, Valerio M, Rojas L (2011). Candida tropicalis fungaemia: incidence, risk factors and mortality in a general hospital. Clin Microbiol Infect.

[22] Magill SS, O’Leary E, Janelle SJ, Thompson DL, Dumyati G, Nadle J (2018). Changes in prevalence of health care-associated infections in US hospitals. N Engl J Med.

[23] Koehler P, Stecher M, Cornely OA, Koehler D, Vehreschild MJGT, Bohlius J (2019). Morbidity and mortality of candidaemia in Europe: an epidemiologic meta-analysis. Clin Microbiol Infect.

[24] Tsay S, Williams S, Mu Y, Epson E, Johnston H, Farley MM (2018). 363. National Burden of Candidemia, United States, 2017. Open Forum Infect Dis.

[25] Guinea J (2014). Global trends in the distribution of Candida species causing candidemia. Clin Microbiol Infect.

[26] Seagle EE, Jackson BR, Lockhart SR, Jenkins EN, Revis A, Farley MM (2022). Recurrent Candidemia: trends and risk factors among persons residing in 4 US States, 2011–2018. Open Forum Infect Dis.

[27] Pfaller MA, Messer SA, Diekema DJ, Jones RN, Castanheira M (2014). Use of Micafungin as a surrogate marker to predict susceptibility and resistance to Caspofungin among 3,764 clinical isolates of Candida by use of CLSI methods and interpretive criteria. J Clin Microbiol.

[28] Fernández-Ruiz M, Puig-Asensio M, Guinea J, Almirante B, Padilla B, Almela M (2015). Candida tropicalis bloodstream infection: incidence, risk factors and outcome in a population-based surveillance. J Infect.

[29] Wang D, An N, Yang Y, Yang X, Fan Y, Feng J (2021). Candida tropicalis distribution and drug resistance is correlated with ERG11 and UPC2 expression. Antimicrob Resist Infect Control.

[30] Kim EJ, Lee E, Kwak YG, Yoo HM, Choi JY, Kim SR, et al. Trends in the epidemiology of Candidemia in intensive care units from 2006 to 2017: results from the Korean National Healthcare-Associated Infections Surveillance System. Front Med (Lausanne). 2020;7. 10.3389/fmed.2020.606976 PMC7773785 33392229

[31] Toda M, Williams SR, Berkow EL, Farley MM, Harrison LH, Bonner L (2019). Population-based active surveillance for culture-confirmed Candidemia—four sites, United States, 2012–2016. MMWR Surveill Summ.

[32] Cuervo G, Garcia-Vidal C, Puig-Asensio M, Merino P, Vena A, Martín-Peña A (2019). Usefulness of guideline recommendations for prognosis in patients with candidemia. Med Mycol.

[33] Pappas PG, Kauffman CA, Andes DR, Clancy CJ, Marr KA, Ostrosky-Zeichner L (2016). Clinical practice guideline for the management of Candidiasis: 2016 update by the Infectious Diseases Society of America. Clin Infect Dis.

